# Genome-Wide Plasma Cell-Free DNA Methylation Profiling Identifies Potential Biomarkers for Lung Cancer

**DOI:** 10.1155/2019/4108474

**Published:** 2019-02-05

**Authors:** Wei Xu, Jun Lu, Qiang Zhao, Jun Wu, Jielin Sun, Baohui Han, Xiaodong Zhao, Yani Kang

**Affiliations:** ^1^School of Biomedical Engineering, Bio-ID Center, Shanghai Jiao Tong University, Shanghai 200240, China; ^2^Department of Pulmonary Medicine, Shanghai Chest Hospital, Shanghai Jiao Tong University, Shanghai 200240, China; ^3^School of Life Sciences, East China Normal University, Shanghai 200240, China; ^4^Shanghai Center for Systems Biomedicine, Shanghai Jiao Tong University, Shanghai 200240, China

## Abstract

As a noninvasive blood testing, the detection of cell-free DNA (cfDNA) methylation in plasma has raised an increasing interest due to diagnostic applications. Although extensively used in cfDNA methylation analysis, bisulfite sequencing is less cost-effective. In this study, we investigated the cfDNA methylation patterns in lung cancer patients by MeDIP-seq. Compared with the healthy individuals, 330 differentially methylated regions (DMRs) at gene promoters were identified in lung cancer patients with 33 hypermethylated and 297 hypomethylated regions, respectively. Moreover, these hypermethylated genes were validated with the publicly available DNA methylation data, yielding a set of ten significant differentially methylated genes in lung cancer, including *B3GAT2*, *BCAR1*, *HLF*, *HOPX*, *HOXD11*, *MIR1203*, *MYL9*, *SLC9A3R2*, *SYT5*, and *VTRNA1*-3. Our study demonstrated MeDIP-seq could be effectively used for cfDNA methylation profiling and identified a set of potential biomarker genes with clinical application for lung cancer.

## 1. Introduction

Lung cancer is one of the major cancer types causing cancer deaths [1]. The unavailability of genetic testing for early cancer diagnosis has been regarded as the major cause of high mortality rate [2, 3]. Since cfDNA with cancer-specific characteristics (such as mutation [4] and epigenetic changes [5]) has been discovered in 1989, it has attracted increasing attention in cancer biology research [6]. Unlike the traditional tissue biopsy characterization with the purpose of prognosis or other clinical assessment, characterizing cfDNA is noninvasive and real-time, which makes cfDNA detection a promising clinical tool for disease surveillance, drug response, and disease recurrence [7–9]. Most of all, cfDNA released from cancer cells can be detected at an early stage, making early diagnosis of cancer possible [10].

Hypermethylation at the tumor suppressor gene promoters plays an important role in the formation and progression of cancer [11]. As a driving force in tumorigenesis, methylation occurs at early stage during cancer formation [12]. Moreover, DNA methylation pattern in plasma cfDNA is similar with that derived from cancer tissue [5, 13]. These observations suggest that cfDNA methylation could serve as a useful biomarker for cancer detection [14].

The methods for characterizing cfDNA methylation could be classified into two categories [15], the qPCR-based methods for detecting individual regions of interest or the deep sequencing-based methods for genome-wide DNA methylation profiling. Although extensively used in cfDNA methylation profiling analysis, bisulfite sequencing is less cost-effective [16]. In contrast, methylated DNA immunoprecipitation coupled with high-throughput sequencing (MeDIP-seq), a genome-wide scale and cost-effective method, has been extensively used in genomic DNA methylome analysis, whereas it is rarely applied in characterizing cfDNA methylation [15]. In this study, we performed cfDNA methylome analysis with MeDIP-seq in lung cancer patients. Our results indicate that MeDIP-seq could be effectively used in cfDNA methylation profiling in cancer patients.

## 2. Materials and Methods

### 2.1. Sample Collection and cfDNA Extraction

Lung cancer patient samples (*n* = 5) were collected from Shanghai Chest Hospital. Healthy individual samples (*n* = 3) were obtained as control. All lung cancer blood samples were obtained from patients with adenocarcinoma (sample information shown in Table 1) and control blood samples were from healthy volunteers (the information not provided). The informed consent was gained from individuals, and the study was approved by the ethics committees of Shanghai Chest Hospital.

All blood samples from the control group and lung cancer patients (∼5 ml) were collected in tubes containing EDTA as anticoagulant, centrifuged for 15 min at 1500 × g. The purified plasma was then stored at -80°C.

The cfDNA was extracted from plasma using QIAamp Circulating Nucleic Acid kit (Qiagen, 55114) according to manufacturer's protocol. The quality of plasma cfDNA was evaluated by Bioanalyzer 2100 (Agilent Technologies).

### 2.2. MeDIP-seq Library Construction and Sequencing

The cfDNA was used for MeDIP-seq library preparation with the method we described previously [17] with some modifications. Briefly, ~50 ng cfDNA was ligated with Illumina adapter using the NEBNext Ultra II DNA Library Prep Kit for Illumina (NEB, E7645) according to manufacturer's instructions. The resulting library was denatured at 95°C for 10 min, incubated immediately on ice for 10 min, and then subjected to immunoprecipitation with 5-Methylcytosine (5-mC) Monoclonal Antibody (Epigentek, A-1014). MeDIP DNA was amplified using Q5 High-Fidelity DNA Polymerase (NEB, M0491) and the amplification products were purified with AMPure XP beads (Beckman). The amplified libraries were evaluated with Bioanalyzer 2100 (Agilent Technologies) and subjected to deep sequencing by Illumina Hiseq 2000.

### 2.3. Data Processing and Analysis

MeDIP-seq raw data reads filtered low-quality reads were mapped to the reference genome (Human hg38) using Bowtie (version 1.0.1) [18]. The MEDIPS analysis package (version 1.24.0) was used for analysis and comparison of DNA methylation datasets of patients and the control [19].

The mapped results were visualized using the Integrative Genomics Viewer (IGV) [20]. Gene ontology (GO) analysis and pathway analysis were performed with clusterProfiler [21] and ingenuity pathway analysis (IPA) (Qiagen).

The 450K methylation array data (Illumina, San Diego, CA, USA) from normal solid lung tissue and patient samples were obtained from TCGA-LUAD project (https://portal.gdc.cancer.gov/projects/TCGA-LUAD). Paired Student's *t*-test was performed between 32 pairs of normal samples and patient samples using R statistical programming language (3.4.3, http://www.R-project.org) on the data processed with beta (*β*) values (proportion of the methylated signal over the total signal), and the hypermethylated target genes with *p* value < 0.05 were selected.

The raw data of MeDIP-seq samples in this study are available in the EMBL database (http://www.ebi.ac.uk/arrayexpress/) under accession number E-MTAB-7163.

### 2.4. Real-Time Quantitative PCR

To validate the methylated regions identified by MeDIP-seq, real-time quantitative PCR (qPCR) assay was carried out with SYBR Green qPCR Master Mix (2X) (Kapa, KK4602) at the StepOnePlus qPCR instrument (Applied Biosystems). The primer sequences are shown in Table S1.

## 3. Results

### 3.1. Whole Genome MeDIP-seq Analysis of cfDNA

The plasma of lung cancer patients (*n* = 5) and healthy controls (*n* = 3) were used in this study. The clinical information of patients is shown in Table 1. The cfDNA was extracted from plasma using the kit (Qiagen).

We observed the size distribution of cfDNA centered on 176 bp with the range of 150–200 bp (Figure S1), which was consistent with the previous study [22].

The MeDIP-seq libraries were constructed with the cfDNA derived from patients (*n* = 5) and the healthy persons (*n* = 3) were treated as control. As expected, all amplified libraries exhibited the main peak of ~298 bp containing the ~120 bp sequencing adapters. Representative size distribution profiles for the libraries are shown in Figure S1. All constructed libraries were subjected to next-generation sequencing.

The cfDNA MeDIP-seq libraries were sequenced with Illumina Hiseq 2000. On average, 30 million and 52 million raw sequenced reads were obtained for patients and controls, respectively (Table 1), of which 53.9% and 52.9% were mapped to the reference genome (Human hg38). After the repetitive reads were filtered out, there are an average 3 million unique reads in the patients and an average 5.2 million unique reads in the controls (Table 2).  Figure S2A shows the distribution of MeDIP signal located in each chromosome.

To validate MeDIP data quality, we performed real-time quantitative PCR analysis for randomly selected methylated genes, including *RARB2*, *ZFP42*, and *PAX9*. The qPCR results indicated that the selected region of each gene was fairly enriched, suggesting that our cfDNA MeDIP-seq result was reliable (Figure S2B).

### 3.2. Distinct cfDNA Methylation Patterns between Patients and Control

**Figure 1:**
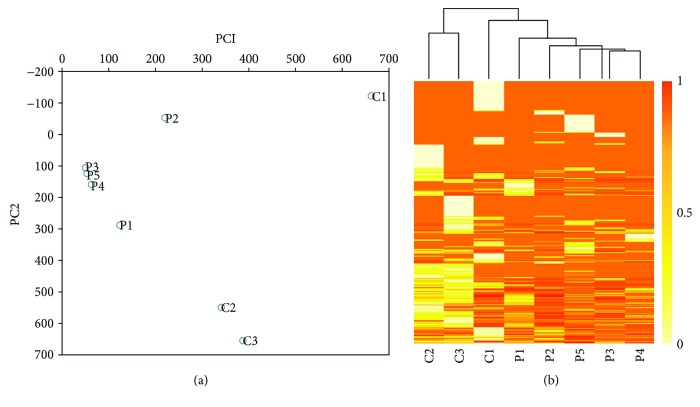
The methylation patterns derived from MeDIP-seq datasets in lung cancer patients and controls. (a) Principal component analysis (PCA) of the methylation profiles of different populations examined. (b) The clustering analysis of the genome-wide methylation profiles in patients and controls.

To examine the overall cfDNA methylation pattern in the patients and the normal, we applied principal component analysis (PCA) to their methylation profiles. Comparing with the control group, we observed the distinct methylation patterns in patients (Figure 1(a)). And the clustering analysis result also indicated that the patients and the control differ in cfDNA methylation patterns (Figure 1(b)).

### 3.3. Differentially Methylated Regions (DMRs) in Lung Cancer Patients

**Figure 2:**
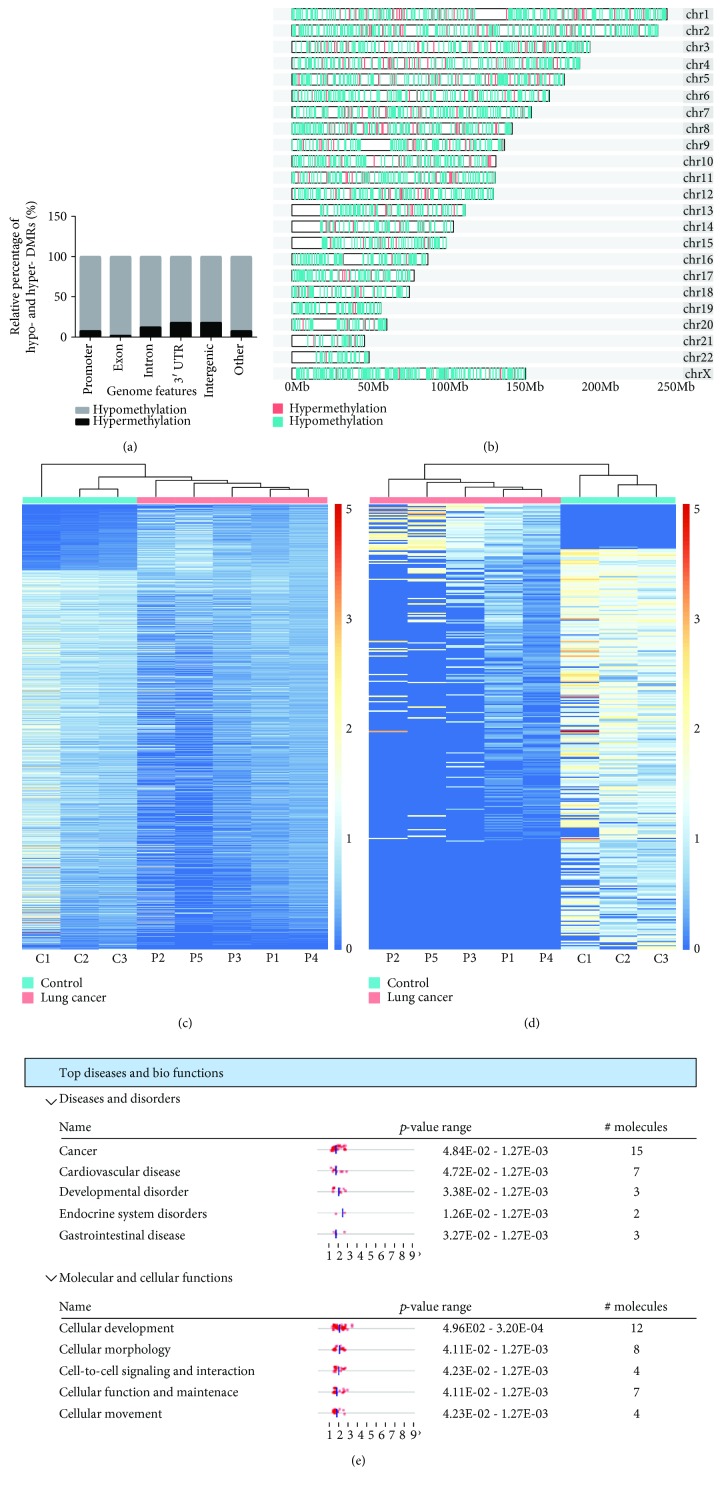
Differentially methylated regions in patients and controls. (a) The distribution of hypermethylated and hypomethylated loci located in exon, intron, promoter, and other genomic features. (b) Representation of the distribution of hypomethylated (green) and hypermethylated (red) regions across patient genomes. (c) Heat map of total 3013 DMRs, including 445 hypermethylated and 2568 hypomethylated. (d) Heat map of DMRs located in promoter regions in both patients and controls. (e) Top diseases and bio functions by IPA analysis for genes with the hypermethylated promoters.

Using the MEDIPS analysis package, 3013 differentially methylated regions (DMRs) were identified in the patients (*p* value < 0.05 and fold change > 2). Moreover, 2568 (85.2%) were hypomethylated and 445 (14.8%) were hypermethylated (Table S2). We examined the genomic distribution of both hypomethylated and hypermethylated DMRs. We found a considerable fraction of DMRs located in intergenic regions (Figure 2(a)). The visual DMR signals of hypomethylation and hypermethylation mapped to whole genome are presented in Figure 2(b). Consistent to what we observed in the overall DNA methylation pattern, these 3013 DMRs also exhibited distinct patterns between patient and the normal (Figure 2(c)).

It is recognized that promoter hypermethylation is associated with cancer development [23]. We next focused on the analysis of DMRs in promoter regions. We found 330 DMRs located in promoter regions (Figure 2(d)), including 33 hypermethylated regions and 297 hypomethylated regions (Table S3). Some genes with hypermethylated promoters have been reported in lung cancer, such as *GAS7* [24], *AQP10* [25], *HLF* [26], and *HOPX* [27].

To understand the biological significance of the genes with hypermethylated promoter in lung cancer patients, we performed gene ontology (GO) analysis. We found that 32 genes derived from 33 hypermethylated DMRs are enriched in tumorigenesis-related GO items, such as oncostatin-M-mediated signaling pathway, negative regulation of gene silencing by miRNA, negative regulation of posttranscriptional gene silencing, cell adhesion, and DNA replication-dependent nucleosome assembly (Table S4). To illustrate the biological processes that these 32 genes were associated with, ingenuity pathway analysis (IPA) software was used and the results are shown in Figure 2(e). The top disease was cancer that involved 15 genes. The top molecular and cellular function was cellular development that involved 12 genes.

### 3.4. Validation of Differentially Methylated Genes with Publicly Available DNA Methylation Data

**Figure 3:**
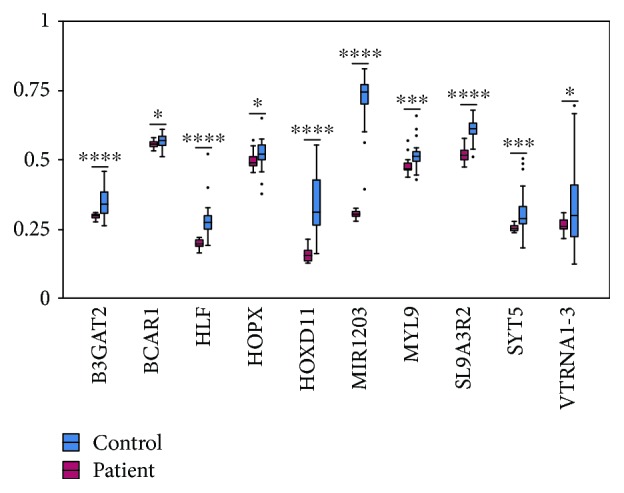
The comparison of methylation level between patients and controls of ten selected genes. ∗ to ∗∗∗∗ represents *p* values of < 0.05, 0.01, 0.001, and 0.0001.

To ask whether the differentially methylated genes identified in our cfDNA study are able to separate the cancer patient from the healthy individuals, we compared the methylation levels of the aforesaid 32 genes in both lung cancer patients (*n* = 36) and healthy individuals (*n* = 36) with publicly available DNA methylation data. We found that there is significant difference (*p* < 0.05) in methylation levels of *B3GAT2*, *BCAR1*, *HLF*, *HOPX*, *HOXD11*, *MIR1203*, *MYL9*, *SLC9A3R2*, *SYT5*, and *VTRNA1*-3 between lung cancer patients and healthy individuals (Figure 3). This result suggests that these ten genes possibly serve as diagnostic biomarkers for lung cancer.

## 4. Discussion

The cfDNA methylation is promising for noninvasive cancer screening and diagnosis [28]. Although extensively used in cfDNA methylation analysis, bisulfite sequencing is less cost-effective. MeDIP-seq, a more cost-effective DNA methylation profiling approach, has not been applied to the study of cfDNA methylation [15, 16]. In the present study [29, 30], we applied MeDIP-seq to characterize the cfDNA methylation pattern in lung cancer patients.

Through MeDIP-seq analysis, we identified 3013 DMRs in cfDNA derived from lung cancer patients, with 2569 (85.3%) hypomethylated and 445 (14.7%) hypermethylated (Figure 2(b)). Such observation was consistent with the well-known phenomenon that cancer genome is featured by the genome-wide demethylation [31]. Methylation at tumor suppressor promoter loci is a driving force in tumorigenesis [32]; we found that only a minority of hypermethylated DMRs was located in promoter regions and a considerable fraction was located in intergenic regions, suggesting that DNA methylation may regulate gene expression in a more complex manner through distant regulatory elements in cancer [33].

To evaluate the clinical potential of 32 hypermethylated genes at promoters identified in cfDNA of patient plasma, we examined the methylation status of this set of genes with DNA methylation data in public database. We found that ten genes exhibited statistically significant difference between lung cancer patients and the normal population, including *B3GAT2*, *BCAR1*, *HLF*, *HOPX*, *HOXD11*, *MIR1203*, *MYL9*, *SLC9A3R2*, *SYT5*, and *VTRNA1*-3 (Figure 3). *HLF* has previously been reported to be methylated in lung cancer [26]. Tumor suppressor *HOPX* inhibits cell proliferation, migration, and invasion in lung cancer [27]. Methylation of *B3GAT2*, a member of the panel as biomarker, has been used for diagnosis in colorectal cancer [34]. These observations suggest that the methylated genes identified in lung cancer plasma could be of potential value in clinical application.

## 5. Conclusions

In brief, our study demonstrated MeDIP-seq could serve as an alternative approach for cfDNA methylation analysis and identified a set of 10 differentially methylated genes as potential biomarkers for clinical application in a lung cancer patient.

## Figures and Tables

**Table 1:** Clinical information for lung patients.

Sample name	Gender	Age	Stage	Histology
P1	Male	50	IIA	Adenocarcinoma
P2	Male	72	IA2	Adenocarcinoma
P3	Female	75	IIB	Adenocarcinoma
P4	Male	73	IIIB	Adenocarcinoma
P5	Female	33	IVB	Adenocarcinoma

Notes: P means cancer patient; number means the patient number.

**Table 2:** Summary statistics of MeDIP-seq data.

Sample	Number of total reads	Number of mapped reads	Mapped read rate	Number of unique reads	Unique read rate
P1	40,691,158	23,618,444	58.0%	7,588,396	32.1%
P2	31,467,734	8,557,316	27.2%	1,389,114	16.2%
P3	35,149,374	25,305,368	72.0%	2,643,030	10.4%
P4	20,488,252	12,351,458	60.3%	2,733,588	22.1%
P5	23,546,814	12,268,278	52.1%	818,628	6.7%
C1	46,505,740	16,686,432	35.9%	2,089,072	12.5%
C2	18,918,360	11,095,194	58.7%	5,085,484	45.8%
C3	91,305,808	58,482,718	64.1%	8,453,424	14.5%

Notes: C means healthy control; P means cancer patient; number means the individual number.

## Data Availability

The raw data of MeDIP-seq in this study are available in the EMBL database (http://www.ebi.ac.uk/arrayexpress/) under accession number E-MTAB-7163.

## Supplementary Materials

Supplementary Materials Figure S1: representative bioanalyzer profiles of cfDNA and MeDIP-seq libraries. Figure S2: MeDIP-seq analysis in lung cancer patients and controls. Table S1: real-time quantitative PCR primer sequences for validating MeDIP-seq enrichment efficiency. Table S2: DMRs identified in cfDNA of lung cancer patient plasma. Table S3: DMRs at promoter regions in cfDNA of lung cancer patient plasma. Table S4: GO annotation terms for the promoter-hypermethylated genes.
