# Supplementary material for: Genome-Wide Plasma Cell-Free DNA Methylation Profiling Identifies Potential Biomarkers for Lung Cancer
Source: Dis Markers. 2019 Feb 5;2019:4108474. doi: 10.1155/2019/4108474 (PMC6379867; doi:10.1155/2019/4108474)
Supplement: Supplementary Materials — Figure S1: representative bioanalyzer profiles of cfDNA and MeDIP-seq libraries. Figure S2: MeDIP-seq analysis in lung cancer patients and controls. Table S1: real-time quantitative PCR primer sequences for validating MeDIP-seq enrichment efficiency. Table S2: DMRs identified in cfDNA of lung cancer patient plasma. Table S3: DMRs at promoter regions in cfDNA of lung cancer patient plasma. Table S4: GO annotation terms for the promoter-hypermethylated genes. [file 4108474.f1.zip › Supplementary Figure S1 Figure S2 Table S1 Table S4.docx]

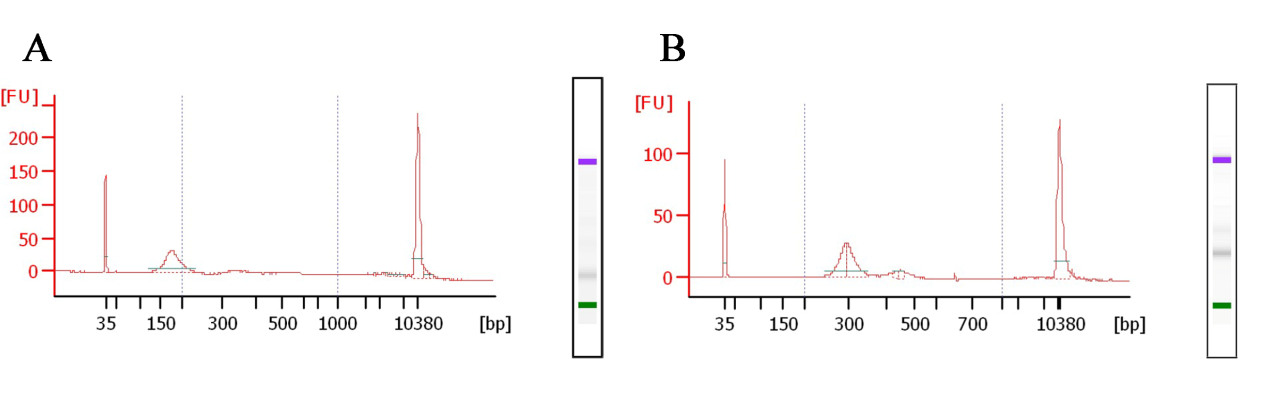


Supplementary Figure S1: Representative bioanalyzer profiles of cfDNA and MeDIP-seq libraries. (A) The fragment size distribution of cfDNA. (B) The fragment size distribution of MeDIP-seq library.


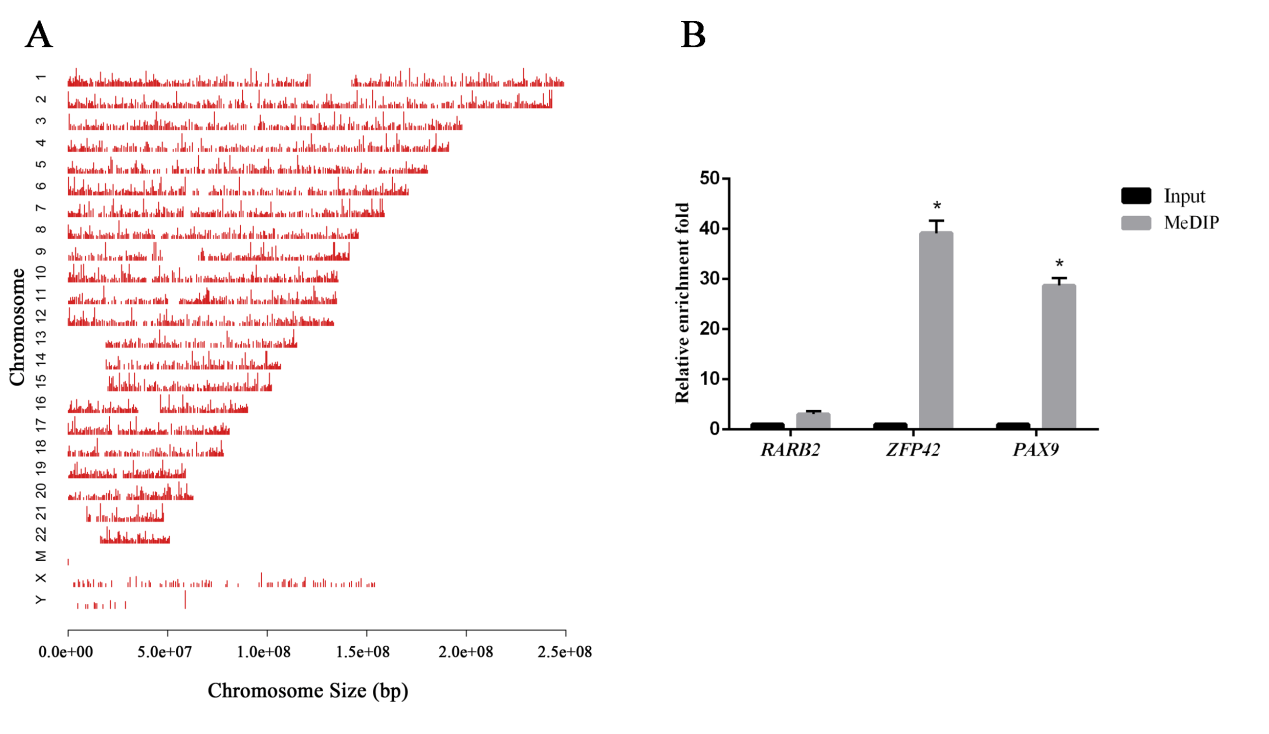


Supplementary Figure S2: MeDIP-seq analysis in lung cancer patients and controls. (A) The density of MeDIP-seq reads across distinct chromosome. (B) Representative real-time quantitative PCR assay for validation of the methylated genomic loci identified by MeDIP-seq analysis. The data were presented as the mean ± SD and * represented p value < 0.05.

| Supplementary Table S1: Real-time quantitive PCR primers sequences for validating MeDIP-seq enrichment efficiency. | |
| --- | --- |
| **Gene name** | **Sequence 5′ to 3′** |
| PAX9-F | GAGGTATGGGGGCTTCTTTG |
| PAX9-R | GAATTCCACCATGTCCCCATTG |
| ZFP42-R | CCATTTGATCGCTTGGGTGTG |
| ZFP42-F | GGAAGAGGTGGAGTGTGAAATGTA |
| RARB2-F | TGGTGAAAATGAACAGTGGACTC |
| RARB2-R | ATCTGAAGCTAGGAGAATCTGGT |
| SEMA6C(control)-F | TCTCGACGTCCACCCTTTTC |
| SEMA6C(control)-R | CTGCTCACTCGAGTCCCCT |

| Supplementary Table S4: GO annotation terms for the promoter-hypermethylated genes. | | |
| --- | --- | --- |
| **ID** | **Description** | **p-value** |
| GO:2000568 | positive regulation of memory T cell activation | 0.002645 |
| GO:0001824 | blastocyst development | 0.002923 |
| GO:0046013 | regulation of T cell homeostatic proliferation | 0.003965 |
| GO:0038165 | oncostatin-M-mediated signaling pathway | 0.005283 |
| GO:0015793 | glycerol transport | 0.007915 |
| GO:0001714 | endodermal cell fate specification | 0.011849 |
| GO:0019755 | one-carbon compound transport | 0.013158 |
| GO:0060965 | negative regulation of gene silencing by miRNA | 0.014464 |
| GO:0060149 | negative regulation of posttranscriptional gene silencing | 0.017073 |
| GO:0060967 | negative regulation of gene silencing by RNA | 0.017073 |
| GO:0030168 | platelet activation | 0.018332 |
| GO:0001916 | positive regulation of T cell mediated cytotoxicity | 0.019674 |
| GO:0033540 | fatty acid beta-oxidation using acyl-CoA oxidase | 0.019674 |
| GO:0035729 | cellular response to hepatocyte growth factor stimulus | 0.019674 |
| GO:0001711 | endodermal cell fate commitment | 0.020973 |
| GO:0048012 | hepatocyte growth factor receptor signaling pathway | 0.020973 |
| GO:0035728 | response to hepatocyte growth factor | 0.022269 |
| GO:0051131 | chaperone-mediated protein complex assembly | 0.023564 |
| GO:0006833 | water transport | 0.024858 |
| GO:0008356 | asymmetric cell division | 0.024858 |
| GO:0042789 | mRNA transcription from RNA polymerase II promoter | 0.024858 |
| GO:0060969 | negative regulation of gene silencing | 0.028728 |
| GO:0001914 | regulation of T cell mediated cytotoxicity | 0.030015 |
| GO:0050906 | detection of stimulus involved in sensory perception | 0.03023 |
| GO:0007155 | cell adhesion | 0.030748 |
| GO:0022610 | biological adhesion | 0.031347 |
| GO:0002526 | acute inflammatory response | 0.031665 |
| GO:0009299 | mRNA transcription | 0.032584 |
| GO:0050974 | detection of mechanical stimulus involved in sensory perception | 0.032584 |
| GO:0042044 | fluid transport | 0.033866 |
| GO:0048011 | neurotrophin TRK receptor signaling pathway | 0.033866 |
| GO:0001825 | blastocyst formation | 0.037703 |
| GO:0002675 | positive regulation of acute inflammatory response | 0.037703 |
| GO:0048791 | calcium ion-regulated exocytosis of neurotransmitter | 0.037703 |
| GO:0050650 | chondroitin sulfate proteoglycan biosynthetic process | 0.037703 |
| GO:0060285 | cilium-dependent cell motility | 0.037703 |
| GO:0001539 | cilium or flagellum-dependent cell motility | 0.038978 |
| GO:0001913 | T cell mediated cytotoxicity | 0.038978 |
| GO:0050716 | positive regulation of interleukin-1 secretion | 0.040252 |
| GO:0070286 | axonemal dynein complex assembly | 0.040252 |
| GO:0002711 | positive regulation of T cell mediated immunity | 0.041524 |
| GO:0006335 | DNA replication-dependent nucleosome assembly | 0.041524 |
| GO:0034723 | DNA replication-dependent nucleosome organization | 0.041524 |
