# Supplementary figures and images for: Genome-Wide Plasma Cell-Free DNA Methylation Profiling Identifies Potential Biomarkers for Lung Cancer
Source: Dis Markers. 2019 Feb 5;2019:4108474. doi: 10.1155/2019/4108474 (PMC6379867; doi:10.1155/2019/4108474)

Chromosome

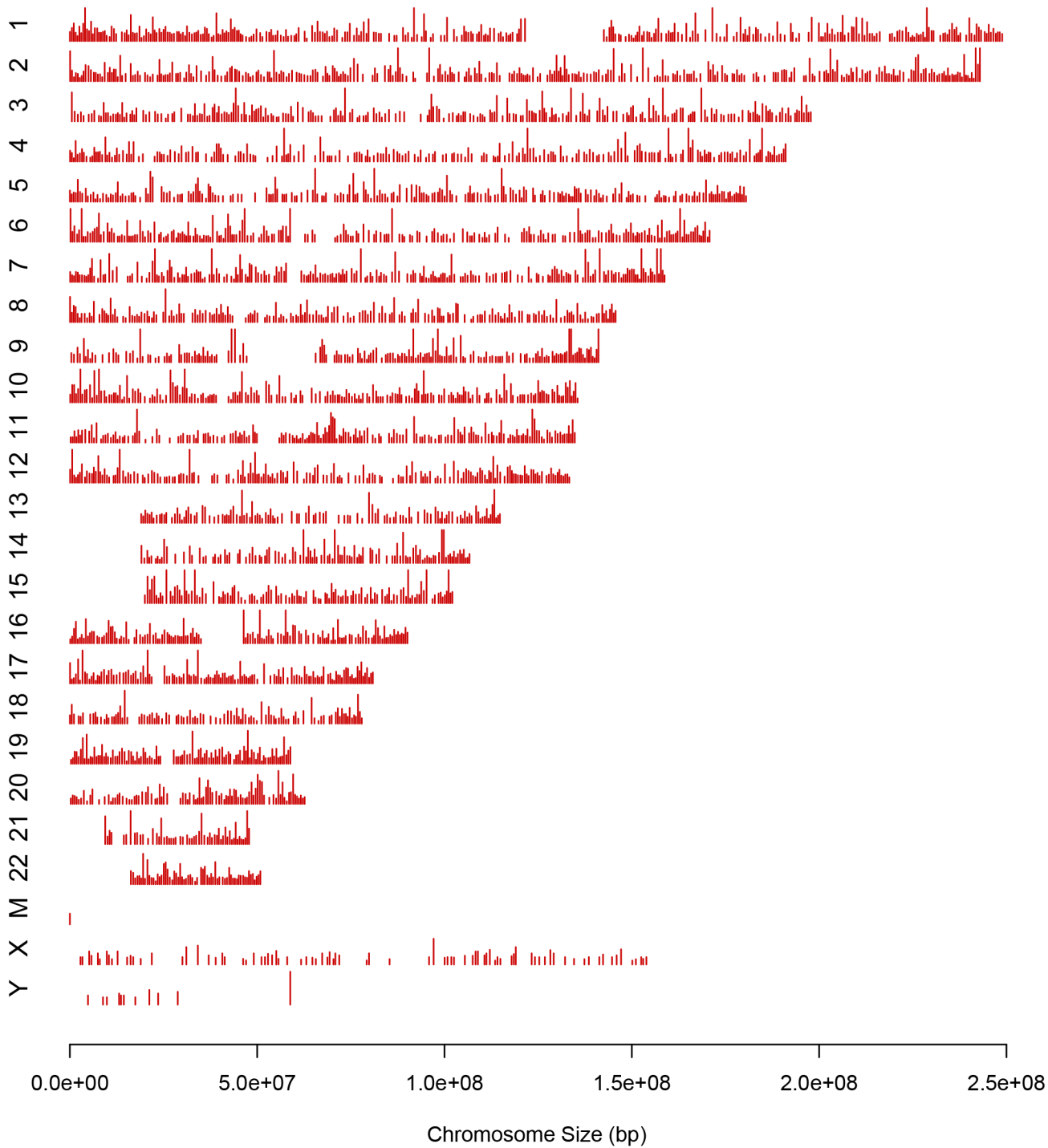

Supplement: Supplementary Materials — Figure S1: representative bioanalyzer profiles of cfDNA and MeDIP-seq libraries. Figure S2: MeDIP-seq analysis in lung cancer patients and controls. Table S1: real-time quantitative PCR primer sequences for validating MeDIP-seq enrichment efficiency. Table S2: DMRs identified in cfDNA of lung cancer patient plasma. Table S3: DMRs at promoter regions in cfDNA of lung cancer patient plasma. Table S4: GO annotation terms for the promoter-hypermethylated genes. [file 4108474.f1.zip › Figure S2A.pdf]
